# Accumulation of major depressive episodes over time in a prospective study indicates that retrospectively assessed lifetime prevalence estimates are too low

**DOI:** 10.1186/1471-244X-9-19

**Published:** 2009-05-08

**Authors:** Scott B Patten

**Affiliations:** 1 Department of Community Health Sciences, University of Calgary, Calgary, Canada

## Abstract

**Background:**

Most epidemiologic studies concerned with Major Depressive Disorder have employed cross-sectional study designs. Assessment of lifetime prevalence in such studies depends on recall of past depressive episodes. Such studies may underestimate lifetime prevalence because of incomplete recall of past episodes (recall bias). An opportunity to evaluate this issue arises with a prospective Canadian study called the National Population Health Survey (NPHS).

**Methods:**

The NPHS is a longitudinal study that has followed a community sample representative of household residents since 1994. Follow-up interviews have been completed every two years and have incorporated the Composite International Diagnostic Interview short form for major depression. Data are currently available for seven such interview cycles spanning the time frame 1994 to 2006. In this study, cumulative prevalence was calculated by determining the proportion of respondents who had one or more major depressive episodes during this follow-up interval.

**Results:**

The annual prevalence of MDD ranged between 4% and 5% of the population during each assessment, consistent with existing literature. However, 19.7% of the population had at least one major depressive episode during follow-up. This included 24.2% of women and 14.2% of men. These estimates are nearly twice as high as the lifetime prevalence of major depressive episodes reported by cross-sectional studies during same time interval.

**Conclusion:**

In this study, prospectively observed cumulative prevalence over a relatively brief interval of time exceeded lifetime prevalence estimates by a considerable extent. This supports the idea that lifetime prevalence estimates are vulnerable to recall bias and that existing estimates are too low for this reason.

## Background

Lifetime prevalence is one of the most frequently reported parameters in psychiatric epidemiology. Lifetime prevalence represents the proportion of the population who have experienced a disorder at some time in their life up to the time of interview. In the case of Major Depressive Disorder, this is the proportion of the population who have experienced a major depressive episode (MDE) but not a manic, hypomanic or mixed episode and who do not have a concurrent psychotic disorder. In Canada, the lifetime prevalence of MDE is 12.2% [1] as determined by a national survey called the Canadian Community Health Survey, Mental Health and Wellbeing (CCHS 1.2) conducted in 2002. Similar values have been reported by a methodologically comparable European study [2]. Higher values have been reported in the US: 16–18% [3,4]. The Canadian study reported a prevalence of past year episodes of 4.8% [1].

Concerns have been expressed about the validity of lifetime prevalence estimates. Andrews et al. followed a cohort that had been admitted to hospital with depressive disorders and found that only about half of these were able to recount their symptoms in a way that was detected by the Composite International Diagnostic Interview (CIDI) 25 years later [5]. The CIDI is the measurement instrument used in each of the lifetime prevalence studies listed above. A tendency to forget about past symptoms or episodes could also explain the otherwise puzzling failure of lifetime prevalence estimates to increase with age [6]. Kruijshaar et al. [7] combined data from the Netherlands Mental Health Survey and Incidence Study (NEMESIS) with the Australian National Study of Mental Health and Wellbeing using a micro-simulation model to explore the role of recall bias. Their results suggested that lifetime prevalence estimates may substantially underestimate the true population values. Discussing some of these observations in a 2005 editorial, Andrews et al. speculated that more than half of the population may experience an episode of MD at some time during their lives [8], far in excess of lifetime prevalence estimates from published cross-sectional studies.

A longitudinal study conducted in Canada provides an opportunity to explore some of these issues. The National Population Health Survey (NPHS) is a general population survey that has included a brief instrument designed to detect past year major depressive episodes, the CIDI short form for major depression (CIDI-SFMD) [9]. In seven interview cycles starting in 1994 and continuing to 2006 the CIDI-SFMD would detect episodes occurring in seven one year intervals. In the absence of recall bias it is reasonable to hypothesize that the proportion of the population reporting an episode during the NPHS should be considerably more than the annual prevalence of MDE, but considerably less than the lifetime prevalence. Canadian lifetime and annual prevalence estimates, see above, also derive from a cross-sectional survey called the Canadian Community Health Survey, Mental Health and Wellbeing (CCHS 1.2). This study used a similar sampling frame and collected data in 2002, however, the CCHS 1.2 used an adaptation of the World Mental Health version of the CIDI [10].

## Methods

The NPHS is a longitudinal study based on a nationally representative community sample assembled by Statistics Canada (Canada's national statistical agency) in 1994. The target population for the NPHS consisted of household residents and did not include homeless persons or residents of institutions. Members of the Canadian Armed Forces, those living on First Nation reserves, and residents of certain remote locations were excluded from the sampling frame. Interviews were conducted in person in 1994 at the beginning of the study, but most of the follow-up interviews have been conducted by telephone. NPHS respondents are interviewed every two years. All of the interviews were carried out by trained and experienced interviewers. Detailed information about the NPHS methods may be found on the Statistics Canada Web page . The NPHS longitudinal cohort included 17,276 participants, but the current analysis was restricted initially to n = 15,254 respondents who were over the age of 12 at the time of the initial 1994 baseline interview. The sample was further restricted to those with complete data collection up to the 2006 interview. Members of the cohort that died, were institutionalized or were lost to follow-up prior to the 2006 interview were excluded. This resulted in the final inclusion of n = 7,457 respondents in the analysis presented here.

The CIDI-SFMD uses a point-based scoring algorithm that incorporates the number of symptom-based criteria fulfilled and the necessity for at least one of two key symptoms (depressed mood and loss of interest or pleasure) in keeping with the Diagnostic and Statistical Manual of Mental Disorders (DSM-IV) [11]. This cut-point also maximized performance of the CIDI-SFMD in a DSM-IIIR-based receiver operator curve analysis carried out during the instrument's development [9]. The CIDI-SFMD does not apply all of the exclusion criteria that are present in DSM-IV. The DSM-IV diagnostic criteria for major depressive episodes have exclusion criteria for episodes that are judged due to the death of a loved one, the effects of a drug or medication or a general medical condition. As the CIDI-SFMD does not apply these exclusions, it may be vulnerable to false positive ratings. Consistent with this idea, experience with the instrument suggests that it may slightly overestimate prevalence [12]. However, any such effects must be modest in magnitude, as the CIDI-SFMD has produced credible estimates during applications in Canada [13,14], the US [15,16] and elsewhere [17]. In the NPHS and related surveys the CIDI-SMFD has consistently replicated the expected pattern and strength of association with demographic and clinical variables [14,18-20]. Furthermore, incidence estimates from the CIDI-SFMD [19,20] are consistent with those of a systematic review of high quality studies by Waraich et al. [21].

All estimates deriving from the NPHS were weighted using sampling weights that account for design features of the study. Because of the complexity of the NPHS sampling strategy, a variance calculation method based on a bootstrap procedure employing 500 replicate sampling weights was employed. The study received ethical approval from the University of Calgary Conjoint Health Research Ethics Board.

## Results

**Figure 1:**
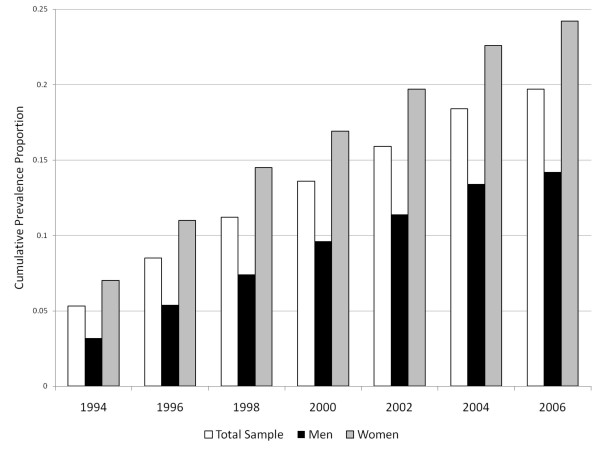
**Cumulative prevalence of MDE in the NPHS 1994 – 2006**.

The pattern of accumulation of MDE is depicted in figure 1. Prevalence at the 1994 interview was 5.3% (95% CI 4.7 – 5.9), and was higher in women (7.0%, 95% CI 6.0 – 8.0) than in men (3.2%, 95% CI 2.4 – 4.0). This 1994 annual prevalence estimate was the highest observed in the seven interviews, with the remainder varying between a low of 4.4% (95% CI 3.8% – 5.0%) in 1998 and a high of 5.0% (95% CI 4.3% – 5.6%) in 2004. Nevertheless, as depicted in figure 1, cumulative prevalence exceeded that of reported Canadian lifetime prevalence. By 2006 the cumulative prevalence was 14.2% in men (95% CI 12.3 – 16.0) and 24.2% in women (95% CI 22.5 – 26.0). In both sexes combined the prevalence was 19.7% (95% CI 18.4 – 21.0). Although the annual prevalence estimates from the NPHS were consistent with estimated annual prevalence in the CCHS 1.2 (4.8%), the cumulative prevalence was nearly twice that of retrospectively assessed lifetime prevalence.

## Discussion

The hypothesis proposed earlier in the analysis: that the cumulative prevalence of MDE over 13 years of follow-up would exceed the annual prevalence of MDE and be lower than lifetime prevalence was not confirmed by the analysis. The accumulation of cases over this relatively brief interval of time substantially exceeded accepted values for lifetime prevalence.

Although the duration of follow-up was 13 years in the sense that data from 1994 to 2006 were used, the CIDI-SFMD items actually only fully covered seven of these years. Many episodes would be expected to begin in one year and end in another, such that the duration of follow-up is best considered greater than seven years but less than thirteen. In any case, the cumulative prevalence over this relatively brief interval far exceeds expectation based on retrospectively assessed lifetime prevalence. Canadian lifetime prevalence from the CCHS 1.2 was 12.2% [1]: only about 60% of the observed cumulative prevalence in the NPHS. The CCHS 1.2 estimate was highly precise (95% CI 11.7 to 12.7), so sampling variability cannot account for the discrepancy reported here. The CCHS 1.2 was conducted in 2002, during same interval covered by the NPHS. Hence, a secular trend in prevalence cannot explain the results either. The most probable explanation is recall bias. However, the analysis presented here cannot directly confirm this possibility. An alternative explanation is that the CIDI-SFMD may generate false positive ratings and thereby overestimate cumulative prevalence. This explanation seems unlikely, however, when one considers that annual prevalence estimates from the NPHS cohort were in close alignment with estimates from other studies, including the CCHS 1.2. A small false positive rate could over time translate into a greater accumulation of episodes during multiple interviews, but an effect of this type seems unlikely to be able to account for the considerable extent to which observed cumulative prevalence exceeded accepted values for lifetime prevalence in this study.

Even though the CIDI-SFMD did not appear to over-identify MDE in the NPHS, it is possible that the annual prevalence identified by the CIDI-SFMD represents a balance between poor specificity and poor sensitivity. In other words, the increased prevalence expected if the instrument produces false positive results could be offset by reduced sensitivity to true MDE, resulting in false negatives. The net result may be that the CIDI-SFMD produces reasonable annual prevalence estimates, but that these estimates include some cases of other conditions such as bereavement or adjustment disorder that, in turn, may accumulate over time in the cohort.

## Conclusion

For the reasons stated above, this study cannot definitively confirm that existing lifetime prevalence estimates are too low. However, it does strongly reinforce a growing literature suggesting that this is the case [5-7]. Accumulating evidence draws the validity of lifetime diagnostic interviews, and their role in psychiatric epidemiologic research, into question.

What is the best solution to these problems? One solution is to determine period prevalence through longitudinal follow-up rather than cross-sectionally, as was done in the NPHS. Unfortunately, this approach can be prohibitively expensive. Another solution is to evaluate current prevalence such that symptoms can be assessed without relying on recall. However, this may reduce the power and achievable precision of studies because a smaller number of respondents would be identified as having an episode. Another option, albeit not one that can address the issue of precision, involves modification of the current approach taken by structured interviews. Instruments such as the CIDI begin with screening questions that refer to lifetime episodes and then explore these episodes in detail, returning to the more recent past through the inclusion of a small number of items asking about the timing of similar recent episodes. This emphasis should perhaps be reversed so that the instruments can obtain a valid and detailed assessment of current mental health status and then assess past history using a smaller number of items.

## Competing interests

The author declares that they have no competing interests.

## Authors' contributions

SBP was the sole author of this study. He designed the study, directed the analysis and wrote the paper.

## Pre-publication history

The pre-publication history for this paper can be accessed here:


